# A multicenter investigator-initiated Phase 2 trial of E7090 in patients with advanced or recurrent solid tumor with fibroblast growth factor receptor (*FGFR*) gene alteration: FORTUNE trial

**DOI:** 10.1186/s12885-022-09949-8

**Published:** 2022-08-09

**Authors:** Yohei Chiba, Kazuki Sudo, Yuki Kojima, Hitomi Okuma, Shinji Kohsaka, Ryunosuke Machida, Masahiko Ichimura, Kenta Anjo, Kazumi Kurishita, Natsuko Okita, Kenichi Nakamura, Ichiro Kinoshita, Masanobu Takahashi, Junichi Matsubara, Hitoshi Kusaba, Kan Yonemori, Masamichi Takahashi

**Affiliations:** 1 Department of Medical Oncology, National Cancer Center Hospital, Tokyo, Japan; 2 Department of Experimental Therapeutics, National Cancer Center Hospital, Tokyo, Japan; 3 Department of International Clinical Development, National Cancer Center Hospital, Tokyo, Japan; 4 Clinical Research Support Office, National Cancer Center Hospital, Tokyo, Japan; 5 Division of Cellular Signaling, National Cancer Center Research Institute, Tokyo, Japan; 6 Division of Clinical Cancer Genomics, Hokkaido University Hospital, Hokkaido, Japan; 7 Department of Medical Oncology, Hokkaido University Hospital, Hokkaido, Japan; 8 Department of Medical Oncology, Tohoku University Hospital, Miyagi, Japan; 9 Department of Clinical Oncology, Kyoto University Hospital, Kyoto, Japan; 10 Department of Hematology, Oncology and Cardiovascular Medicine, Kyushu University Hospital, Fukuoka, Japan; 11 Department of Neurosurgery and Neuro-Oncology, National Cancer Center Hospital, Tokyo, Japan

**Keywords:** Fibroblast growth factor receptor, Gene fusion, Mutation, Amplification, Precision medicine

## Abstract

**Background:**

Aberrant fibroblast growth factor receptor (FGFR) signaling can substantially influence oncogenicity. Despite that FGFR gene abnormality is often detected by cancer genome profiling tests, there is no tumor-agnostic approval yet for these aberrations. E7090 (tasurgratinib) is an orally available selective tyrosine kinase inhibitor of FGFR1-3. Specific FGFR alterations were previously reported to be highly sensitive to E7090 based on a high-throughput functional evaluation method, called mixed-all-nominated-mutants-in-one (MANO) method, narrowing down the most promising targets. This trial was focused on the alterations identified by the MANO method and was performed under the nationwide large registry network for rare cancers in Japan (MASTER KEY Project).

**Methods/Design:**

This single-arm Phase 2 trial was designed to evaluate the safety and efficacy of E7090 in patients with advanced or recurrent solid tumors harboring FGFR alterations. Three cohorts were set based on the type of FGFR alterations and the results of MANO method. A maximum of 45 patients will be enrolled from 5 institutions over 2.5 years. E7090 will be administered once daily as an oral single agent in 28-day cycles. The primary endpoint is the objective overall response rate; whereas, the secondary endpoints include progression-free survival, overall survival, disease control rate, safety, duration of response, and time to response. Ethics approval was granted by the National Cancer Center Hospital Certified Review Board. Patient enrollment began in June 2021.

**Discussion:**

A unique investigator-initiated multicenter Phase 2 trial was designed based on the results of preclinical investigation aiming to acquire the approval of E7090 for solid tumors harboring FGFR gene alterations. The findings may serve as a novel model for the development of tumor-agnostic molecular targeted therapies against rare genetic abnormalities.

**Trial registration:**

Japan Registry of Clinical Trial: jRCT2031210043 (registered April 20, 2021) ClinicalTrials.gov: NCT04962867 (registered July 15, 2021).

## Background

Research on targeted gene therapy, also known as “precision medicine,” is being conducted extensively worldwide. Particularly, cancer genome medicine is being promoted. Although cancer genome profiling tests using next-generation sequencing (NGS) show that approximately 40 to 60% of patients have genetic abnormalities that could serve as therapeutic targets, the percentage of patients who practically receive biomarker-driven and molecular targeted therapy remains low at about 10% [1, 2]. To promote the development of new treatments, we initiated an industry-academia collaborative project called the Marker Assisted Selective Therapy in Rare Cancers: Knowledge Database Establishing Registry Project (‘MASTER KEY Project’) in Japan. This is a master protocol study that consists of 2 parts: a prospective registry component that collects biomarker and clinical data from patients with rare cancers, and a clinical trial component that conducts biomarker-directed or non-biomarker-directed clinical trials [3]. In the literature, personalized treatment using a genomic biomarker has shown a higher response rate (RR) and prolonged median progression-free survival (PFS) [4, 5].

The fibroblast growth factor (FGF)/FGF receptor (FGFR) signaling pathway plays multiple roles in the regulation of cellular functions, affecting cell differentiation, migration, proliferation, and survival [6]. The FGF proteins are a large family of multifunctional peptide growth factors that bind to and activate a family of 4 FGFRs [7]. FGFRs with genetic abnormalities, such as gene fusion, somatic mutation, or amplification, are present in 4.1 to 5.1% of human cancers [2, 8]. Most molecular alterations promote multiple steps of carcinogenesis in *FGFR* oncogene-addicted cells; thereby, increasing cell proliferation, angiogenesis, and drug resistance [9]. The in vivo and in vitro oncogenic potential studies of these aberrations suggest a potent sensitivity to FGFR inhibitors [10, 11]. Several FGFR inhibitors are currently approved by the U.S. Food and Drug Administration for the treatment of urothelial or cholangiocarcinoma, including erdafitinib, [12] pemigatinib, [13] and infigratinib [14]. However, in Japan, pemigatinib is the only drug approved for the treatment of unresectable cholangiocarcinoma patients with *FGFR2* gene fusion.

E7090 (tasurgratinib) is a novel orally administered tyrosine kinase inhibitor that selectively inhibits FGFR1, 2 and 3; this drug was discovered and developed by Eisai's Tsukuba Research Laboratories [15]. A first-in-human Phase 1 study of E7090 in patients with advanced solid tumors has been conducted in Japan (ClinicalTrials.gov: NCT02275910); wherein, one dose-limiting toxicity of Grade 3 aspartate aminotransferase (AST)/alanine aminotransferase (ALT) increase was observed in the 180-mg once-daily dosing group; therefore, the recommended dose was determined to be 140-mg once daily. Of the 24 patients treated in the dose-escalation part, one achieved a partial response and 7 patients achieved stable disease [16]. Preliminary results in the expansion part of this Phase 1 study showed the activity of E7090 with an overall response rate (ORR) of 83.3% (5/6) and 11.1% (1/9) in patients with cholangiocarcinoma harboring an *FGFR*2 gene fusion and in those with gastric cancer harboring either *FGFR*2 gene amplification or FGFR2 protein high expression, respectively. The observed median PFS was 8.26 months in patients with cholangiocarcinoma and 2.58 months in those with gastric cancer [17]. A global Phase 2 study targeting *FGFR*2 gene fusion in unresectable advanced or metastatic cholangiocarcinoma is ongoing (ClinicalTrials.gov: NCT 04,238,715).

While E7090 exhibited the promising responses in patients with cholangiocarcinoma, the transforming activity and sensitivity of 160 nonsynonymous *FGFR* mutations and 10 fusion genes to eight FGFR tyrosine kinase inhibitors (TKIs) using a high-throughput functional assay using the mixed-all-nominated-mutants-in-one (MANO) method was evaluated [11, 18, 19]. The FGFR TKIs showed anti-proliferative activities against proteins encoded by wild-type *FGFR*s and their gene fusions; several hotspot mutants were relatively resistant to these TKIs. Importantly, the efficacy of FGFR inhibitors for each variant was different among inhibitors, and several hotspot mutations were specifically sensitive to E7090 [11]. Therefore, inhibition of FGFR signaling by E7090 can be an attractive therapeutic option for several tumor types.

The essence of cancer precision medicine is to provide individualized treatment proposals based on genomic diagnosis. And it is obviously essential to have a drug delivery system as an exit strategy. In Japan, due to the limited number of patients, some situations existed where investigational drugs for a rare population were approved by merely a single-arm Phase 2 study that mainly evaluates response rates [20]. This study described here is a novel attempt to evaluate a new drug with a tumor agnostic approach by incorporating the MANO method on rare gene alterations detected by NGS. The authors intend to discuss the results of this study with the Agency for consideration of an approval.

## Methods/design

### Aim

To evaluate the efficacy and safety of E7090 in advanced or recurrent solid tumors with *FGFR* gene alterations (including fusion, mutation, and amplification).

### Study setting

**Fig. 1:**
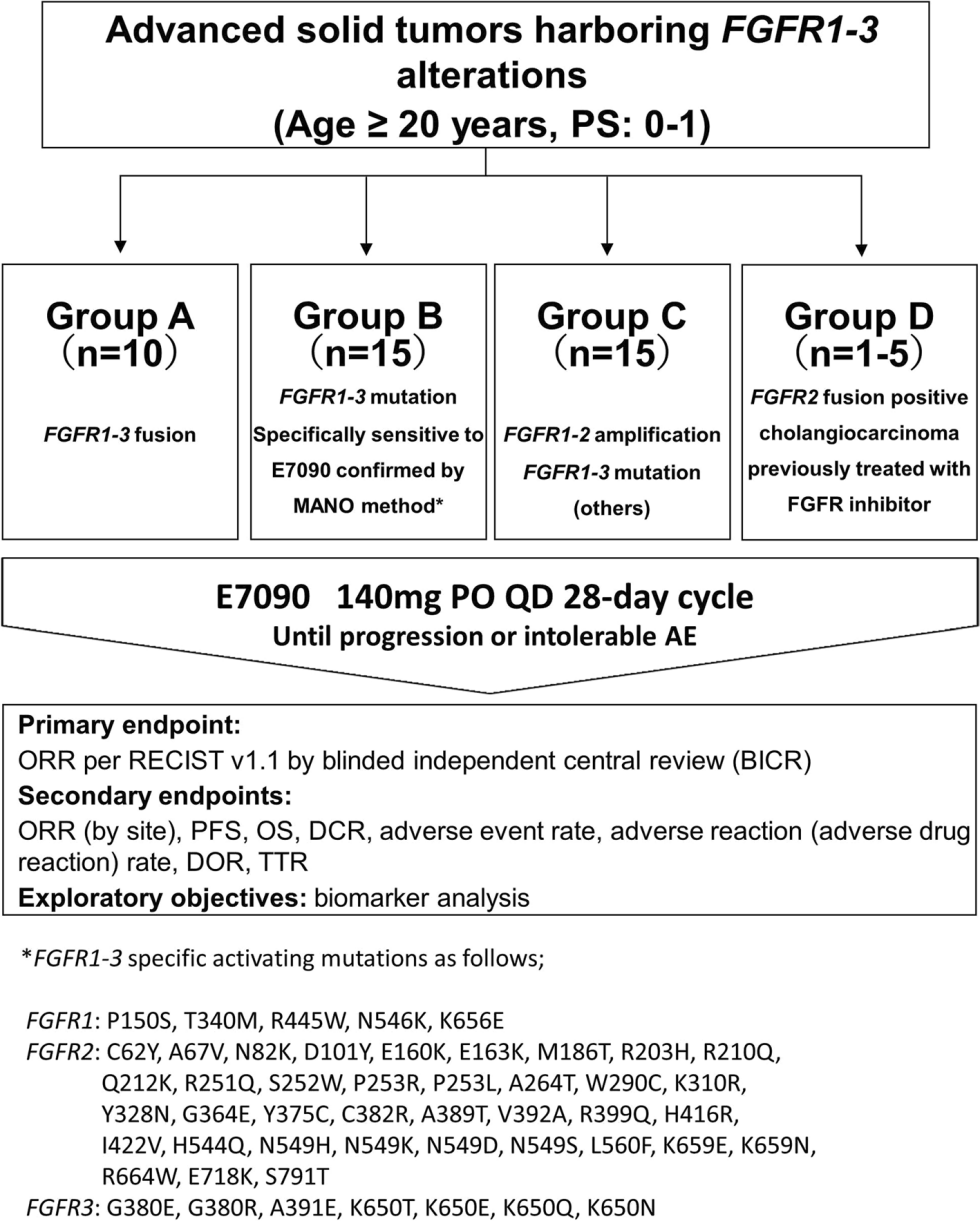
Flow diagram of the FORTUNE protocol. A total of 41 to 45 patients will be enrolled; it is expected that the number of patients in Group A, B, C, and D will be approximately 10, 15, 15, and 5, respectively. Group A includes patients with advanced solid tumors harboring *FGFR1-3* fusion. Group B includes patients with advanced solid tumors harboring *FGFR1-3* mutation suggesting that the efficacy of E7090 is promising by MANO method. Group C includes patients with advanced solid tumors harboring *FGFR1-3* mutation other than what is included Group B and/or *FGFR1, 2* amplification. Group D includes patients with cholangiocarcinoma harboring *FGFR2* gene fusion who received prior selective FGFR inhibitors other than E7090. E7090 is administered once daily as an oral single agent in 28-day cycles

This study was a single-group, open-label, investigator-initiated, multicenter Phase 2 basket study (Fig. 1).

### Endpoints

The primary endpoint was ORR, defined as the combined incidence of complete response (CR) and PR, confirmed no less than 4 weeks after the criteria for response were first met, based on response evaluation criteria in solid tumors (RECIST) v1.1. ORR was confirmed by blinded independent central review (BICR).

The secondary endpoints were ORR (confirmed by a local site investigator), PFS, overall survival (OS), disease control rate (DCR), safety, duration of response (DOR), and time to response (TTR). Safety endpoints included the adverse event (AE) rate and adverse reaction (adverse drug reaction) rate. The severity of AEs was evaluated by the investigator according to the Japan Clinical Oncology Group (JCOG) Japanese translation of the Common Terminology Criteria for Adverse Events version 5.0 (CTCAE v5.0-JCOG).

### Eligibility criteria

1. Participant with histologically or cytologically confirmed metastatic, unresectable, or recurrent solid tumor who agreed to provide an archival tumor sample, a residual biopsy sample, or a fresh tumor biopsy sample
2. Participant experiencing ineffective treatment or who is intolerant to initial treatment, or for which standard treatment is no longer available
3. Participant with an *FGFR* gene alteration detected by NGS panel*, who fell under one of the categories of the following groups:
  Group A: *FGFR1-3* fusion
  Group B: *FGFR1-3* specific activating mutations as below;
  *FGFR1*: P150S, T340M, R445W, N546K, K656E
  *FGFR2*: C62Y, A67V, N82K, D101Y, E160K, E163K, M186T, R203H, R210Q, Q212K, R251Q, S252W, P253R, P253L, A264T, W290C, K310R, Y328N, G364E, Y375C, C382R, A389T, V392A, R399Q, H416R, I422V, H544Q, N549H, N549K, N549D, N549S, L560F, K659E, K659N, R664W, E718K, S791T
  *FGFR3*: G380E, G380R, A391E, K650T, K650E, K650Q, K650N
  Group C: *FGFR1-3* activating mutation not applicable to group B or *FGFR1, 2* amplification
  Group D: Participant with cholangiocarcinoma harboring *FGFR2* gene fusion who previously received a selective FGFR inhibitor other than E7090 and demonstrated progressive disease or resistance.
4. Participant is age less than20 years old.
5. Participant whose Performance Status (Eastern Cooperative Oncology Group [ECOG]) is 0–1.
6. Participant(s) with non-primary CNS tumors; the ones who had at least 1 lesion of ≥ 1.0 centimeter (cm) in the longest diameter for a non-lymph node or ≥ 1.5 cm in the short-axis diameter for a lymph node that was serially measurable according to RECIST v1.1, using computerized tomography/magnetic resonance imaging (CT/MRI).
7. Participant with primary CNS tumors should fulfill the following criteria:
  - Karnofsky performance status (KPS) ≥ 70
  - Prior treatment, including radiation and/or chemotherapy, as recommended or appropriate for CNS tumor type
  - ≥ 1 site of bi-dimensionally measurable disease (confirmed by MRI and evaluable by response assessment in neuro-oncology [RANO] criteria), with at least one measurable lesion ≥ 1 cm in each dimension and noted on more than one imaging slice
  - Must be neurologically stable based on neurological examination for 7 days prior to enrollment.
8. Participant with corrected calcium ≤ 10.1 mg/dL: corrected calcium (mg/dL) = serum calcium (mg/dL) + (4-serum albumin).
9. Participant with phosphate ≤ 4.6 mg/dL.
10. Participants who required treatment washout period from the last day of prior treatment until enrollment of this trial, was as follows:
  - Antibody and other investigational drugs: ≥ 28 days
  - Prior chemotherapy, surgical therapy, radiation therapy: ≥ 21 days (≥ 90 days from the date of the last radiation therapy for primary CNS tumors)
  - Endocrine therapy, immunotherapy, small-molecule targeted therapy: ≥ 14 days

* Testing refers to insurance-applied NGS tests including FoundationOne®CDx Cancer Genome Profile/OncoGuide™ NCC Oncopanel System.

### Exclusion criteria

1. Patients with brain, subdural, or leptomeningeal metastases
2. Patients with primary CNS tumor located in either cerebellum, brainstem, spinal cord, pituitary gland, optic nerve, or olfactory nerve
3. Patients who are positive for human immunodeficiency virus (HIV) antibody, HBs antigen, or HCV antibody (patients with positive HCV antibody but no detectable HCV-RNA were not excluded)
4. Patients who are negative for HB antigen, positive for HBs antibody or HBc antibody, and positive for HBV-DNA quantification (not excluded if HBV-DNA is below detection sensitivity)
5. Patient with Child-Pugh score of B or C
6. Patients with pericardial effusion, pleural effusion, or ascites requiring treatment
7. Patients with presence of any of the following ocular diseases:
  - Grade 2 or higher corneal disorders
  - Active retinopathy (eg, age-related macular degeneration, central serous chorioretinal disease, retinal tear)
8. Participants who require drugs that strongly inhibit or induce the activity of metabolizing enzyme cytochrome P450 (CYP) 3A
9. *FGFR* gatekeeper mutation: *FGFR1* V561, *FGFR2* V564/565, *FGFR3* V555/557, *FGFR4* V550
10. Patients with any of the following coexisting driver gene abnormalities:
  - Genetic mutations (excluding VUS): *KRAS*, *NRAS*, *EGFR*, or *BRAF* V600
  - Gene translocations of *ALK*, *ROS1*, or *NTRK*

### Treatment methods

Participants received E7090 140 mg tablets orally once daily (QD) in a 28-day treatment cycle until disease progression, development of unacceptable toxicity, participant requested to discontinue, withdrawal of consent, or study termination occurred. E7090 was administered at least 1 h before breakfast or at least 2 h after meals, and no food was ingested for 1 h after administration. The investigational drug used in this study was a 35-mg tablet. Dose reduction was in the order of 105 mg, 70-mg, and 35-mg.

### Follow-up

Participants were evaluated at scheduled visits over the following study periods: Screening, Treatment, and Follow-up. Evaluations during the screening period were conducted within 28 days before the administration of the first dose of the study drug. Procedures conducted during the screening period that were performed within 4 days before first dose of E7090 may also have been used as the baseline evaluation and did not need to be repeated, unless otherwise specified. Participants were followed-up every 6 months following the initial enrollment date of the first participant. Follow-up was to continue until death or a maximum of 1 year following enrollment of the last participant.

### Efficacy evaluations

Tumor assessments will be primarily based on the “Guidelines for Evaluating Treatment Effectiveness in Solid Tumors (RECIST Guidelines); Revised RECIST Guidelines version 1.1” [21]. A CT scan or MRI will be performed every 8 weeks (± 7 days) up to 24 weeks, every 12 weeks (± 7 days) after 25 weeks, and every 16 weeks (± 7 days) after 49 weeks from the start of protocol treatment until disease progression, death, withdrawal of informed consent, or treatment discontinuation. RANO criteria are widely used internationally in the field of brain tumors [22]. In this study, we also adopted the definition of the RANO criteria for tumor assessment in patients with primary central nervous system (CNS) tumors to compare with other clinical trials for primary brain tumors and to retain reference values that may serve as historical controls for future studies.

### Safety evaluations

The safety evaluation includes ECOG performance status (± KPS]), vital signs (blood pressure, pulse rate, body temperature, oxygen saturation [SpO_2_]), laboratory evaluations, urinalysis, 12-lead electrocardiogram, AE collection, and ophthalmic examination. Safety follow-up will be performed on Day 30 (+ 7 days) after the final dose, or the start of post-treatment, whichever comes first. The CTCAE v5.0-JCOG will be used in this study to grade clinical and laboratory AEs.

### Sample size calculation and statistical methods

The sample sizes in Groups A, B, and C were determined based on an exact test. A true ORR of ≤ 5% and a true ORR of ≥ 30% were considered as null and alternative hypotheses, respectively. Under a one-sided alpha error of 5%, considering the feasibility of case accumulation, the required number of patients in Groups A, B, and C were 10 (power 62%), 15 (87%), and 15 (87%), respectively. The sample size in group D was not based on statistical assumptions, and 5 patients can be enrolled in this as the maximum. ORR and DCR were calculated, and their 95% confidence intervals were estimated based on the Clopper-Pearson method. PFS and OS were evaluated using the Kaplan–Meier method, and 95% confidence intervals for median survival time and survival rate were estimated using the Brookmeyer and Crowley method and Greenwood’s formula, respectively. The proportion of patients who experienced AEs was also estimated. For each group, all treated populations were defined as those who received at least one drug dose. All statistical analyses for efficacy and safety endpoints were based on this population and were performed using SAS version 9.4 (SAS Institute, Inc., Cary, NC, USA).

## Discussion

A single-arm basket study for the rare genetic abnormality, FGFR gene alteration, was designed under the nationwide network for rare cancers called MASTER KEY project. Utilizing this platform, we collated patient data across Japan through each cancer genome core hospital designated by the Ministry of Health, Labour and Welfare. This is one of the strengths of this study, as it allows the accumulation of cases with rare genetic abnormalities in a relatively short period of time.

Another feature of this study is the unique grouping of less frequent genetic abnormalities using expectations for drug response detected by MANO methods. It is suggested that the contribution of genetic abnormalities of FGFR to the carcinogenesis of solid tumors increases in the order of fusion gene, gene mutation, and gene amplification. This is also supported by the fact that the response rates in early clinical trials of FGFR inhibitors other than E7090 for FGFR gene aberrations were also higher for gene fusion (-45%), mutation (-17.6%), and amplification (-6.2%), in that order [23–27]. As the contribution of each FGF/FGFR gene pathway to activation is higher, inhibiting that signaling pathway is expected to have a higher therapeutic effect. In this study, patients were divided into groups A to C according to the expected efficacy of E7090 for each genetic abnormality based on robust results of preclinical study. Group A is for fusion, which has the highest response rate in the previous report. The mutations in group B that are found to be oncogenic by the MANO assay and have similar efficacy compared with the fusion genes are considered to be a group with a high contribution to carcinogenesis in the pathway inhibited by E7090. For group C, it is unclear whether gene amplification contributes to carcinogenesis, and for mutations other than group B, the contribution to carcinogenesis in the pathway inhibited by E7090 is estimated to be lower than that of group B based on the MANO method. Therefore, the design of this study was to collectively evaluate populations that were thought to be similar in 2 aspects: the expected efficacy and contribution to carcinogenesis of each genetic abnormalities.

The limitation of the study is that due to the small sample size, each cohort does not necessarily include all types of cancers and genetic abnormalities However, the efficacy of each individual genetic abnormality is not necessarily required for regulatory approval in Japan. The authors intend to discuss the results of this study with the PMDA, the Japanese regulatory authority, as there is precedent for approvals based on small samples in indications with a rare incidence, a clear benefit is observed and there is a high unmet need.

Available treatment options and the effectiveness of further treatments after the end of standard of care are very limited in most of cancers. This study and findings may serve as a novel model for the development of tumor-agnostic molecular targeted therapies for patients who have rare genetic abnormalities.

## Data Availability

Data sharing is not applicable to this article as no datasets were generated or analysed during the creating process of current protocol.

## Abbreviations

AE: Adverse Event
BICR: Blinded Independent Central Review
BOR: Best Overall Response
CNS: Central Nervous System
CR: Complete Response
CT: Computed Tomography
CTCAE: Common Terminology Criteria for Adverse Events
ECOG: Eastern Cooperative Oncology Group
FGF: Fibroblast Growth Factor
FGFR: Fibroblast Growth Factor Receptor
HBc: Hepatitis B core
HBs: Hepatitis B surface
HBV: Hepatitis B Virus
HCV: Hepatitis C Virus
HIV: Human Immunodeficiency Virus
JCOG: Japan Clinical Oncology Group
KPS: Karnofsky Performance Status
MANO method: Mixed-all-mutants-in-one method
MRI: Magnetic Resonance Imaging
NGS: Next-Generation Sequencing
ORR: Overall Response Rate
OS: Overall survival
PFS: Progression Free Survival
PS: Performance Status
QD: Quaque die
RANO: Response assessment in neuro-oncology
RECIST: Response Evaluation Criteria in Solid Tumors
TTR: Time To Response
